# MS: Wip1 suppresses angiogenesis through the STAT3-VEGF signalling pathway in serous ovarian cancer

**DOI:** 10.1186/s13048-022-00990-6

**Published:** 2022-05-10

**Authors:** Sheng Yin, Lina Yang, Yiyan Zheng, Rongyu Zang

**Affiliations:** 1 Department of Gynaecologic Oncology, Ovarian Cancer Program, Zhongshan Hospital, Fudan University, Shanghai, China; 2 Department of Obstetrics and Gynecology, Zhongshan Hospital, Fudan University, Shanghai, China; 3 Department of Obstetrics and Gynecology, Shanghai Fifth People’s Hospital, Fudan University, Shanghai, China

**Keywords:** Wip1, Serous ovarian cancer, Angiogenesis, STAT3, Inflammation

## Abstract

Multifaceted functions of the so-called “oncogene” Wip1 have been reported in a previous study, while its actual role remains to be explored in serous ovarian cancer (SOC). In this study, by performing bioinformatic analysis with a public database and immunohistochemical staining of Wip1 in tumour tissue from SOC, we concluded that decreased expression of Wip1 was associated with a higher rate of tumour metastasis and platinum-based therapy resistance and increased ascites volume, which led to poorer prognosis in SOC patients. We also found that overexpression of Wip1 in SKOV3 cells decreased the levels of several cytokines, including VEGF, by secretome profiling analysis, and Wip1 overexpression suppressed angiogenesis both in vitro and in vivo. Mechanistic studies indicated that overexpression of Wip1 decreased the expression of VEGF at both the protein and mRNA levels and that the inhibitory effect was mediated by dephosphorylation of STAT3 at Ser727. Our study uncovered the role of Wip1 in SOC and provides a novel therapeutic strategy for suppressing angiogenesis.

## Introduction

Ovarian cancer is the most lethal gynaecological carcinoma and is usually diagnosed at advanced stages with extensive abdominal metastases [1]. Although radical cytoreductive surgery and chemotherapy prolong survival, most patients suffer symptomatic recurrence of disease and chemotherapy resistance [2]. As cancers are composed of several different subtypes with different landscapes, individual therapy has been found to be important [3].

Wip1 (wild-type p53-induced phosphatase 1) inactivates p38, p53, ATM, Chk2, and γ-H2AX and returns cells to homeostasis when DNA damage repair (DDR) is completed [4–6]. In addition to the important function of Wip1 in the DDR, Wip1 has been identified as an oncogene because Wip1 overexpression or *PPM1D* amplification is found in many types of carcinomas, such as breast [7], gastric [8], and lung [9] carcinomas, and high expression of Wip1 may cause tumorigenesis by suppressing the function of the tumour suppressor p53.

Serous ovarian cancer (SOC) is the most common ovarian cancer subtype, and The Cancer Genome Atlas (TCGA) database shows that approximately 90% of high-grade SOC patients harboured p53 mutations [10]. There have been few reports on the function of Wip1 in SOC.

In our previous study [11], we found that Wip1 suppressed invasion and migration by negatively regulating p-ATM, p-Akt, and Snail in both ovarian cancer cells and xenograft animal models. In clinical specimens, we also found that the expression of Wip1 was negatively correlated with p-ATM, p-Akt, and Snail expression, which suggested that Wip1 may serve as a tumour suppressor in SOC. As many studies have demonstrated the multifaceted function of Wip1 and its p53-independent characteristics [12–14], we aimed to determine the role of Wip1 in SOC.

In this study, we found that Wip1 may act as a tumour suppressor in SOC by performing bioinformatic analysis, and the results were confirmed by immunohistochemical (IHC) staining of Wip1 in tumour tissues. We also found that decreased expression of Wip1 was correlated with chemoresistance and ascites volume. Finally, we demonstrated that Wip1 negatively regulates angiogenesis through the STAT3-VEGF signalling pathway.

## Methods and materials

### Bioinformatic analysis

The Human Protein Atlas (https://www.proteinatlas.org) was employed to analyse the clinical outcomes of patients with different Wip1 expression levels in different types of cancer, including colorectal cancer, liver cancer, and ovarian cancer. The prognostic significance of *PPM1D* mRNA expression was assessed in the online database Kaplan–Meier Plotter (www.Kmplot.com). Patients with ovarian cancer were split into high and low expression groups according to the automatically selected best cut-off mRNA expression value. Patient survival outcomes, including overall survival (OS) and progression-free survival (PFS), were assessed between the two groups. The hazard ratios (HRs), 95% confidence intervals (CIs), and log-rank *P* values for each corresponding *PPM1D* gene were determined.

### Tissue microarray construction and immunohistochemistry

A total of 122 patients diagnosed with high-grade SOC with positive p53 staining were selected from our previous study [15]. The expression of Wip1 was determined based on the percentage of positive cancer cells and the staining intensity as previously described [11]. Clinical data included patient age at diagnosis, FIGO (International Federation of Gynecology and Obstetrics) stage, preoperative CA125 value and intraoperative findings. Follow-up was performed for all patients to calculate the PFS, OS and TFI (treatment-free interval). Patients with TFI ≤ 6 months were considered “chemoresistant”.

### Cytokine array and data acquisition

Cytokines in culture media were detected and analysed using Human L507 Array slides (AAH-BLG-1–2, RayBiotech, Norcross, GA) by H-Wayen Biotechnologies (Shanghai, China). In brief, media without FBS was harvested from SKOV3/Vector and SKOV3/Wip1 cells after incubation for 48 h. In general, the concentrations of CMs were measured with a BCA protein assay kit (ThermoFisher Scientific Inc., USA) using a VICTOR Nivo multimode plate reader (PerkinElmer, USA). The Human L507 Array slides were dried for 2 h at room temperature and incubated with 800 μL blocking solution at room temperature for 30 min. After decanting the blocking buffer from each subarray, 400 μL diluted culture media was added and incubated overnight at 4 ℃. After decanting the samples, all arrays were washed 3 times with 800 μL of 1 × wash buffer I. Then, the slides were incubated with Cy3-conjugated streptavidin solution and rinsed with 1 × wash buffers I and II. The results were assessed using a GenePix 4000 B Scanner (Axon Instrument, USA) and analysed with the GenePix Pro 6.0 program (Axon Instrument, USA). Global normalization of the fluorescent spots was conducted to reduce noise and variation among the samples.

### Quantitative real-time PCR (qRT–PCR)

Total RNA was extracted using TRIzol (Invitrogen, USA) according to the manufacturer’s protocol and was reverse transcribed into cDNA using the PrimeScript RT Reagent Kit with gDNA Eraser (Takara, Japan). The expression of mRNA was measured using FastStart Universal SYBR Green Master Mix (Rox) (Roche, Switzerland) on the Applied Biosystems 7500 Real-Time PCR System (Thermo Fisher Scientific Inc., USA). GAPDH was utilized as an internal control. The relative expression of target genes was evaluated with the 2^−ΔΔct^ method. The assay was performed three times in triplicate.

The primer pairs for cDNA amplification were as follows:

- 5’-CACACAGGATGGCTTGAAGA-3’ (forward) and
- 5’-AGGGCAGAATCATCACGAAG-3’ (reverse) for *VEGFA*;
- 5’-TTGAGGTCAATGAAGGGGTC-3’ (forward) and
- 5’-GAAGGTGAAGGTCGGAGTCA-3’ (reverse) for *GAPDH*; and
- 5’-GCCAGAACTTCCCAAGGAAAG-3’ (forward) and
- 5’-GGTTCAGGTGACACCACAAATTC-3’ (reverse) for *PPM1D.*

### Tube formation assay

For the tube formation assay, 70 μL Matrigel (Corning, NY) per well was added to 96-well plates and incubated at 37 °C for 60 min to allow gelation to occur. HUVECs/Vector or HUVECs/Wip1 were added to the top of the gel at a density of 40 000 cells per well. Then, the cells were incubated at 37 °C with 5% CO_2_ overnight, and images were captured under an Olympus inverted microscope equipped with a CCD camera (Olympus, Japan). The degree of tube formation was analysed by counting the number of complete tubes.

### Western blot analysis

Cells were collected and lysed using RIPA lysis buffer (Beyotime, China). After quantification using a BCA Protein Assay Kit (Beyotime, China), the samples were mixed with loading buffer, and 30 μg protein samples were analysed. The following primary antibodies were used: antibodies against Wip1 (SC-H300, Santa Cruz Biotechnology, USA), VEGFA (AF0312, Beyotime Biotechnology, China), CD31 (ab28364, abcam, UK), HIF-1α (ab51608, abcam, UK), β-actin (MABT825, Sigma Aldrich, USA), STAT3 (CST-9139), p-STAT3 Ser727 (CST-9134), and p-STAT3 Tyr705 (CST-9131) were purchased from Cell Signaling Technology Inc. (USA). Immunoreactivity was measured according to standard procedures.

### Statistics

SPSS software package version 19.0 was used for all statistical analyses. The chi-square test was used to evaluate the association of Wip1 expression with different clinical characteristics. Survival analysis was performed using the Kaplan–Meier method. For in vitro data, statistical analyses were performed using unpaired Student’s t tests with GraphPad Prism V8.0 software (La Jolla, CA, USA), and a *p*-value < 0.05 was considered statistically significant. All data analysed were from three separate experiments. The significance of the data is represented as ^*^ *p* < 0.05, ^**^ *p* < 0.01, and ^***^ *p* < 0.001.

## Results

### Wip1 may be a tumour suppressor in serous ovarian cancer

**Fig. 1:**
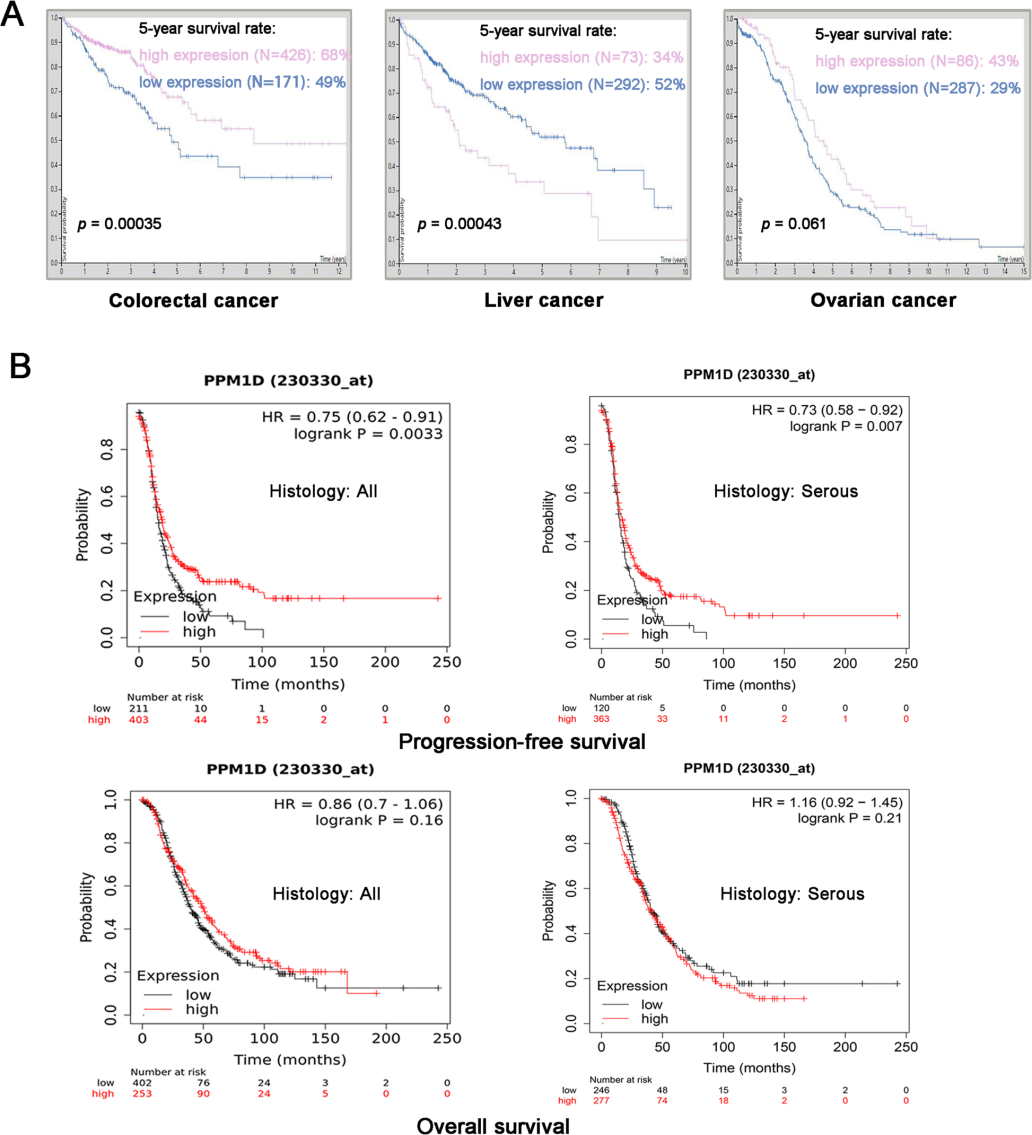
**A** The correlation of Wip1 expression with patient prognosis in colorectal cancer, liver cancer and ovarian cancer according to the Human Protein Atlas database. **B** The prognostic value of *PPM1D* in ovarian cancer patients according to the Kaplan–Meier Plotter database

To identify the role of Wip1, we performed bioinformatic analysis with the publicly available Human Protein Atlas database. As shown in Fig. **1**A, Wip1 was a prognostic marker in both colorectal cancer and liver cancer. A total of 597 colorectal cancer patients were included in the HPA database analysis, of whom 171 and 426 patients had low and high Wip1 expression, respectively. The Kaplan–Meier curves showed that patients with high expression of Wip1 had favourable prognosis in colorectal cancer (the 5-year survival rate was 68% and 49% in the high and low expression groups, respectively; the *p* value was 0.00035). In the liver cancer analysis, a total of 365 patients were included, of whom 292 and 73 patients had low and high Wip1 expression, respectively. The Kaplan–Meier curves showed that patients with high expression of Wip1 had unfavourable prognosis in liver cancer (the 5-year survival rate was 34% and 52% in the high and low expression groups, respectively; the *p* value was 0.00043). In the ovarian cancer analysis, a total of 373 patients were included, of whom 287 and 86 patients had low and high Wip1 expression, respectively. Although Wip1 was not a prognostic marker (the *p* value was 0.061), patients with ovarian cancer harbouring high Wip1 expression showed a favourable 5-year survival rate compared with those with low expression (43% vs. 29%).

Then, Kaplan–Meier Plotter was applied to analyse the relationship between the mRNA expression of *PPM1D* and patient prognosis in overall ovarian carcinoma and serous-subtype ovarian carcinoma. The results suggested that patients with low *PPM1D* mRNA expression had significantly poorer PFS and relatively poorer OS, although the *p* value was not significant (as shown in Fig. 1B). These results suggested that *PPM1D* may be a tumour suppresser in serous ovarian cancer.

### Wip1 expression is positively associated with patient outcome

**Fig. 2:**
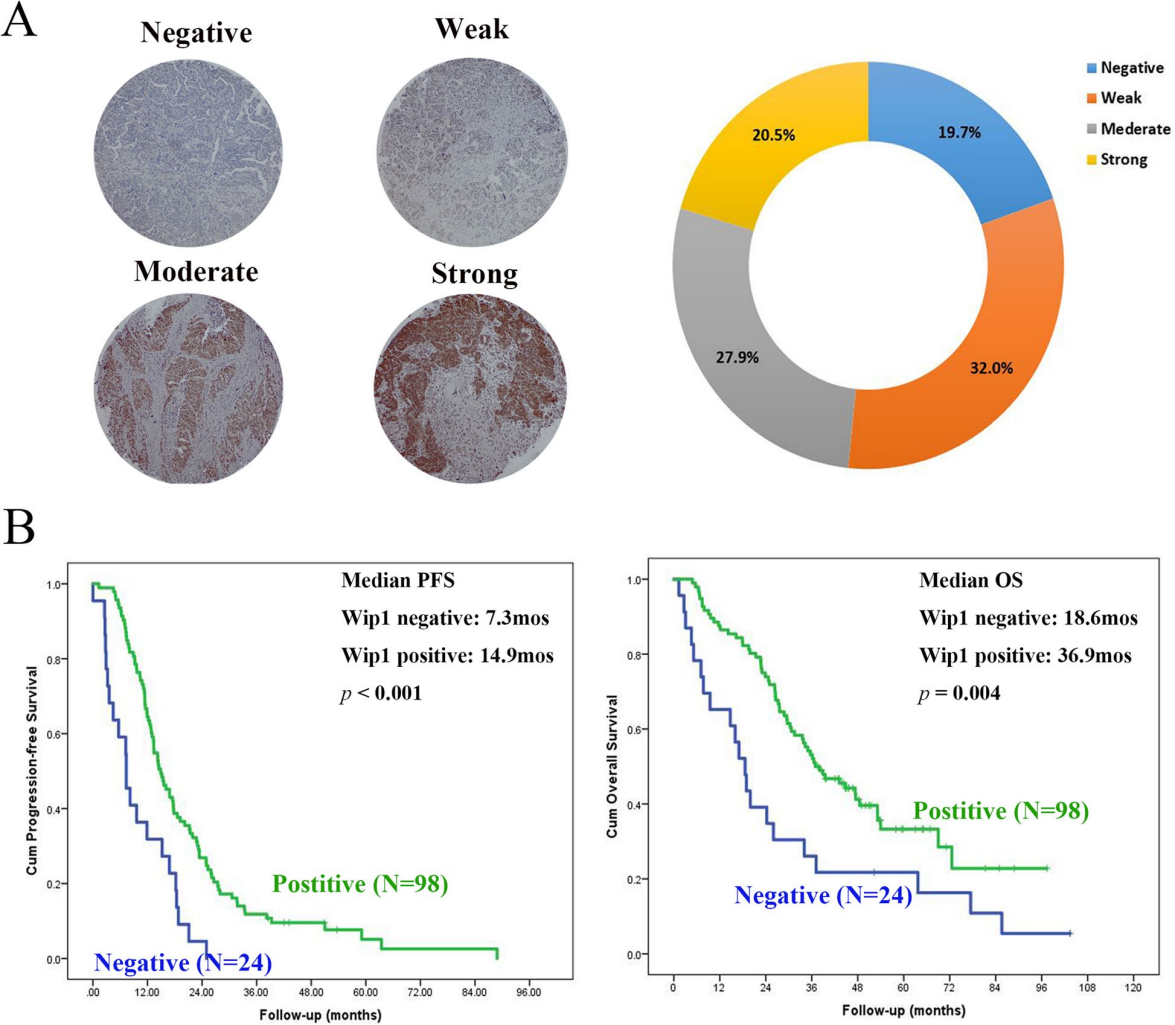
**A** Representative images of serous ovarian cancer biopsies containing negative, weak, moderate and strong staining of Wip1. **B** Kaplan–Meier progression-free survival (PFS) and overall survival (OS) curves (log-rank tests) of patients with negative and positive staining of Wip1

To analyse the association of Wip1 expression and clinicopathological parameters, 122 serous ovarian carcinoma tissues with positive p53 staining were identified and included in the analysis. As shown in Fig. 2A, in the 122 patients with advanced ovarian cancer, Wip1 staining was negative in 24 (19.7%) and positive in 98 (80.3%) patients. The positive group consisted of 39 (32.0%), 34 (27.9%) and 25 (20.5%) patients with weak, moderate and strong expression, respectively. Then, we compared the clinical characteristics between patients with negative and positive expression of Wip1. As shown in Table 1, the baseline patient characteristics in the Wip1-negative group and positive group were well balanced, including age, FIGO stage and median preoperative serum CA125. Patients with negative Wip1 staining showed a higher volume of ascites (*p* = 0.014), more bowel mesenteric metastasis (*p* = 0.04) and more diaphragm metastasis (*p* = 0.008). Furthermore, patients in the negative Wip1 expression group were more likely to be chemoresistant (*p* = 0.005). The Kaplan–Meier curves shown in Fig. 2B indicated that patients with negative Wip1 staining had a significantly poorer survival than those with positive staining of Wip1, with a median PFS of 7.3 mos and 14.9 mos in the Wip1-negative and positive groups, respectively (*p* < 0.001), and the median OS was 18.6 mos and 36.9 mos in the Wip1-negative and -positive groups, respectively (*p* = 0.004).

**Table 1:** Clinical characteristics of patients with negative and positive staining of Wip1

	Wip1 staining		*P* value
	Negative (*n* = 24)	Positive (*n* = 98)	
Median age	57.0 years	57.5 years	0.684
FIGO stage			
III	24 (100%)	90 (91.8%)	
IV	0	8 (8.2%)	0.353
Median preoperative serum CA125	1827.5 U/mL	1507 U/mL	0.478
Ascites volume			
≤ 500 mL	1 (4.2%)	27 (27.6%)	
> 500 mL	23 (95.8%)	71 (72.4%)	0.014
Bowel mesenteric metastasis			
No	6 (25.0%)	48 (49.0%)	
Yes	18 (75.0%)	50 (51.0%)	0.04
Diaphragm metastasis			
No	3 (12.5%)	41 (41.8%)	
Yes	21 (87.5%)	57 (58.2%)	0.008
TFI			
≤ 6 mos	17 (70.8%)	35 (35.7%)	
> 6 mos	5 (20.8%)	59 (60.2%)	
NA	2 (8.3%)	4 (4.1%)	0.005

Abbreviations: *FIGO* International Federation of Gynecology and Obstetrics, *TFI* treatment-free interval, *NA* not available

### Wip1 suppressed VEGF expression and angiogenesis in serous ovarian cancer cells

**Fig. 3:**
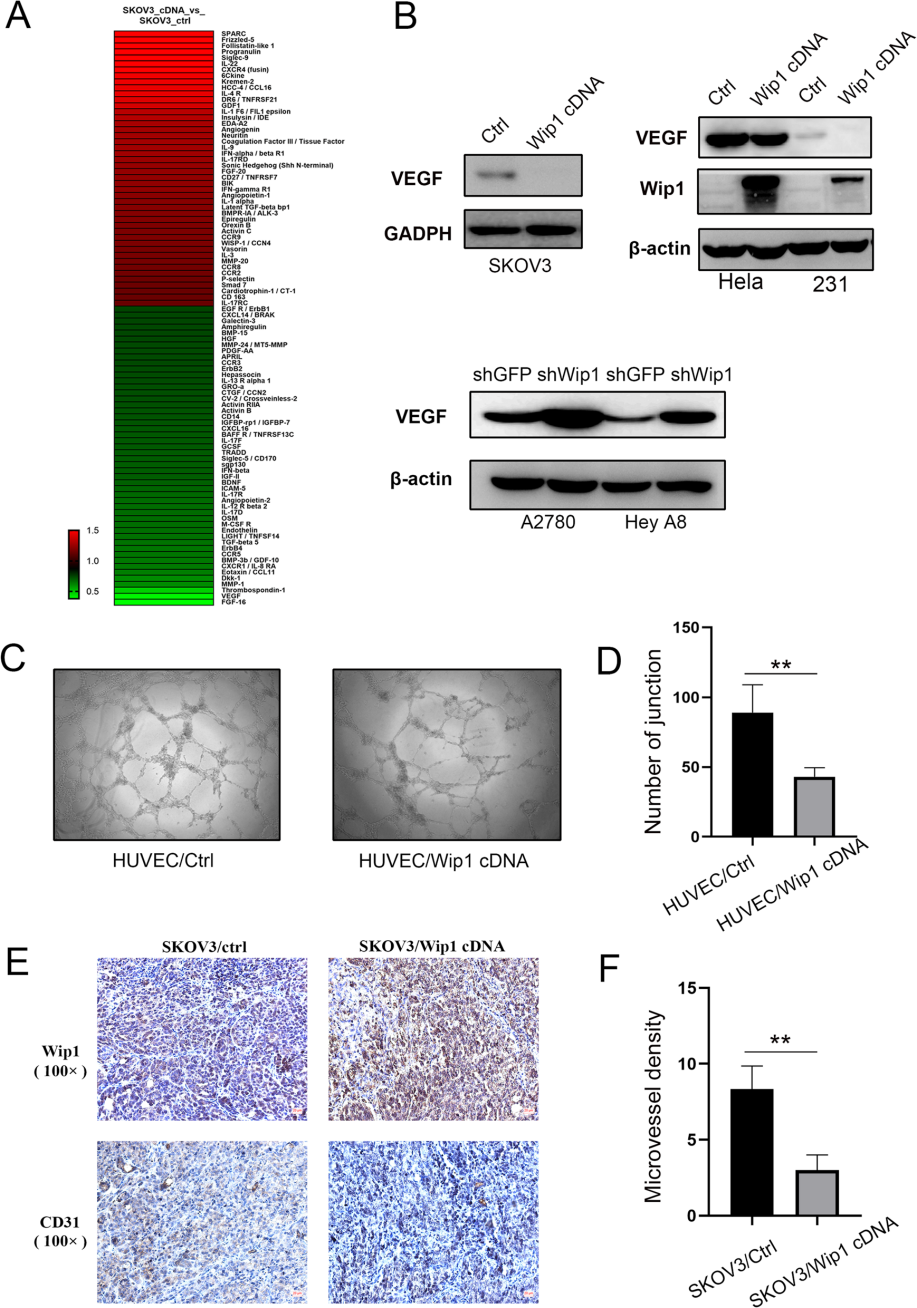
**A** Secretome profiling of differentially expressed cytokines from the culture media of SKOV3/Vector and SKOV3/Wip1 cells. **B** Overexpression of Wip1 decreased VEGF in SKOV3, HeLa and MDA-MB-231 cells, while knockdown of Wip1 increased VEGF in A2780 and HEY A8 cells. **C** Representative images of the tube formation of HUVECs/Vector and HUVECs/Wip1. **D** Statistical analysis of tube branch points. **E** Immunohistochemical staining of Wip1 and CD31 in peritoneally disseminated nodules in nude mice injected with SKOV3/Vector and SKOV3/Wip1 cells. **F** Statistical analysis of microvessel density

We demonstrated that the expression of Wip1 was negatively correlated with the volume of ascites in both animal experiments [11] and serous ovarian cancer patients. A high-density antibody array containing 507 different proteins, including cytokines and growth factors, was applied to analyse culture media harvested from SKOV3/Vector and SKOV3/Wip1 cells. Analysis of the array dataset by significance analysis of microarrays (SAM) revealed significant changes in the levels of a number of serum proteins after Wip1 overexpression. As shown in **Fig. ****3****A**, the three most upregulated proteins were SPARC, Frizzled-5 and Follistatin-like 1 (the natural logarithm-transformed mean fold changes from baseline were 1.91, 1.74 and 1.59, respectively), and the most downregulated proteins included FGF-16, VEGF and thrombospondin-1 (the natural logarithm-transformed mean fold changes from baseline were 0.38, 0.40 and 0.49, respectively).

Since VEGF is a key mediator of angiogenesis in cancer, we next verified whether Wip1 negatively regulated VEGF expression. We tested VEGF expression in both Wip1-overexpressing and Wip1-knockdown cells by western blotting. As shown in Fig. 3B, Wip1 overexpression suppressed VEGF expression in SKOV3 cells. Interestingly, we also found that the expression of VEGF was downregulated when Wip1 was transiently upregulated in HeLa (cervical adenocarcinoma) and MDA-MB-231 (breast cancer) cells. On the other hand, Wip1 knockdown significantly upregulated VEGF expression in both A2780 and Hey A8 cells (Fig. 3B). A tube formation assay was also employed to detect the effect of Wip1 on angiogenesis. The angiogenic ability of HUVECs in the Wip1 overexpression group was significantly decreased compared to that of HUVECs in the control group (Fig. 3C and 3D). To further verify whether Wip1 suppressed angiogenesis in vivo, microvessel density (MVD) was analysed via immunohistochemical staining of CD31 in tumour tissue slices from SKOV3/Vector and SKOV3/Wip1 intraperitoneal tumour models. As shown in Fig. 3E and 3F, the SKOV3 Wip1 overexpression group showed decreased CD31 staining, implying decreased MVD.

### Wip1 inhibited VEGF expression by dephosphorylating STAT3

**Fig. 4:**
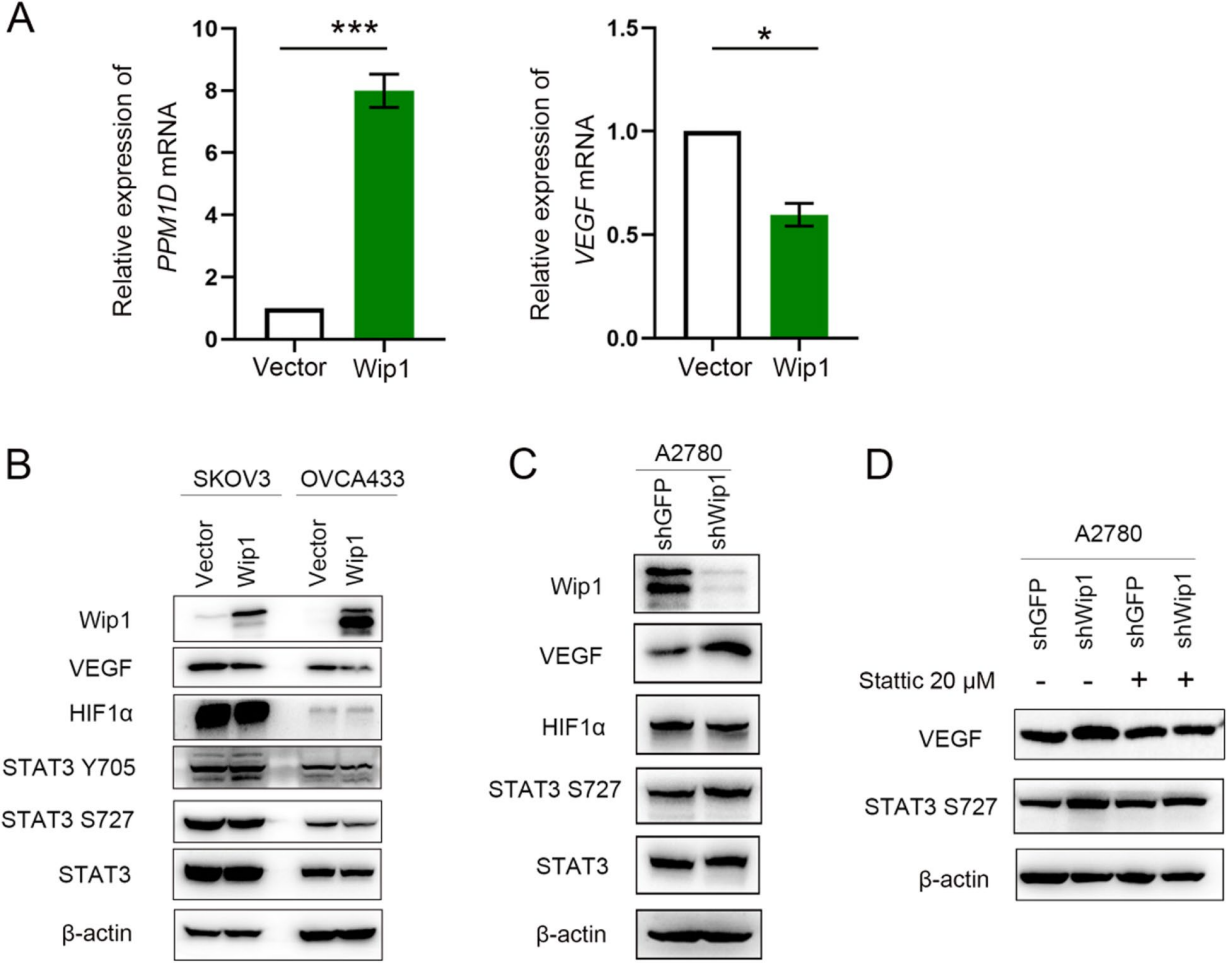
**A** Relative expression of *PPM1D* and *VEGF* mRNA in SKOV3/Vector and SKOV3/Wip1 cells. **B** Overexpression of Wip1 decreased the expression of VEGF and p-STAT3 (S727) but had no effect on the expression of HIF-1α, STAT3 and p-STAT3 (Y705). **C** Knockdown of Wip1 increased the expression of VEGF and p-STAT3 (S727) in A2780 cells. **D** The STAT3 inhibitor Stattic (20 μM) weakened the effect of VEGF upregulation in A2780/shWip1 cells

To investigate the molecular mechanism by which Wip1 regulates VEGF expression, we first tested whether the transcription level of *VEGF* was changed by qRT–PCR. As shown in Fig. 4A, Wip1 overexpression downregulated *VEGF* mRNA in SKOV3 cells. These results suggested that Wip1 regulated VEGF expression at both the protein and transcription levels. We next tested whether upstream binding transcription factors of VEGF, including HIF1α and STAT3, were changed with western blotting. As shown in Fig. 4B, Wip1 overexpression did not change the expression of HIF1α, STAT3 or p-STAT3 (Tyr705). However, we found that the expression of p-STAT3 (Ser727) was significantly downregulated in Wip1-overexpressing cells (both SKOV3 and OVCA433 cells). Moreover, p-STAT3 (Ser727) expression was upregulated when Wip1 was knocked down in A2780 cells (Fig. 4C). To further verify whether p-STAT3 S727 was involved in Wip1-inhibited VEGF expression, we utilized the STAT3 inhibitor Stattic to inhibit the phosphorylation of STAT3. As shown in Fig. 4D, inhibition of STAT3 abrogated the upregulation of VEGF induced by Wip1 knockdown. These results indicated that Wip1 negatively regulates VEGF expression by dephosphorylating STAT3 at the Ser727 site.

## Discussion

Our study showed that Wip1 downregulation predicts poor prognosis in serous ovarian cancer, and Wip1 downregulation was correlated with an increased rate of chemoresistance and increased ascites volume. In addition, our results suggested that Wip1 suppresses angiogenesis both in vivo and in vitro, and the p-STAT3/VEGF signalling pathway may exert important functions on Wip1-mediated angiogenesis.

Previous studies have demonstrated that by dephosphorylating p53, Wip1 plays an oncogenic role in several carcinomas, including breast, liver, and ovarian clear cell carcinoma. However, several studies have indicated that Wip1 may be a tumour suppressor in p53-negative tumours. Goloudina et al. showed that Wip1 inhibitors may be ineffective in tumours exhibiting loss of functional p53. Furthermore, Wip1 overexpression increased the sensitivity of tumour cells with inactive p53 to cisplatin both in vivo and in vitro [12, 16]. Clausse et al. performed bioinformatics analysis of two cohorts of patients with colon cancer, including patients with tp53-wild-type and tp53-mutated colon cancer [17]. The results showed that the effect of Wip1 overexpression depended on the p53 status. High expression of *PPM1D* in wild-type p53 tumours correlated with poor prognosis but with good prognosis in p53-negative tumours. In our study, we concluded that negative Wip1 expression was correlated with chemoresistance in patients with serous ovarian cancer, which was in accordance with a previously published study.

Both inflammation and EMT are correlated with tumour aggressiveness and poor prognosis in cancer patients. Inflammatory cytokines can activate transcription factors that drive EMT [18, 19]. In our previous study, we identified that Wip1 suppresses metastasis in ovarian cancer through AKT/snail-mediated EMT. In this study, we showed that Wip1 may also act as an inflammation and angiogenesis suppressor in serous ovarian cancer. Previous studies have defined a functional role of Wip1 in inflammation. Chew et al. identified that Wip1 knockdown results in increased NF-kappaB function and enhanced inflammation in vivo [20]. Studies also showed that Wip1 expression was negatively correlated with neutrophil production of inflammatory cytokines, including TNF-α, IL-6 and IL-1β, in sepsis patients, which was mediated by the p38 MAPK-STAT1 and NF-kappaB pathways [21–23]. Overall, Wip1 was found to be a negative regulator of EMT, inflammation and angiogenesis in serous ovarian cancer.

In conclusion, our study revealed that decreased expression of Wip1 was correlated with a higher rate of tumour metastasis and chemoresistance and increased ascites volume, and Wip1 negatively regulated angiogenesis through the STAT3-VEGF signalling pathway. Increasing and not suppressing the expression of Wip1 may be a therapeutic strategy in SOC.
