# Correction: Wip1 suppresses angiogenesis through the STAT3-VEGF signalling pathway in serous ovarian cancer

**DOI:** 10.1186/s13048-022-01017-w

**Published:** 2022-07-10

**Authors:** Sheng Yin, Lina Yang, Yiyan Zheng, Rongyu Zang

**Affiliations:** 1 Department of Gynaecologic Oncology, Ovarian Cancer Program, Zhongshan Hospital, Fudan University, Shanghai, China; 2 Department of Obstetrics and Gynecology, Zhongshan Hospital, Fudan University, Shanghai, China; 3 Department of Obstetrics and Gynecology, Shanghai Fifth People’s Hospital, Fudan University, Shanghai, China

**Correction: J Ovarian Res 15, 56 (2022)**

10.1186/s13048-022-00990-6

In the original publication of this article [1], there were mistakes in the article.

1) The word "MS:" in the title refers to manuscript, which should be deleted in the formal document. The authors opted to correct the title to “Wip1 suppresses angiogenesis through the STAT3-VEGF signalling pathway in serous ovarian cancer”.

2) Fig. 3 was wrongly uploaded during the process of article production, which should be replaced by the new figure below.

**Fig. 3:**
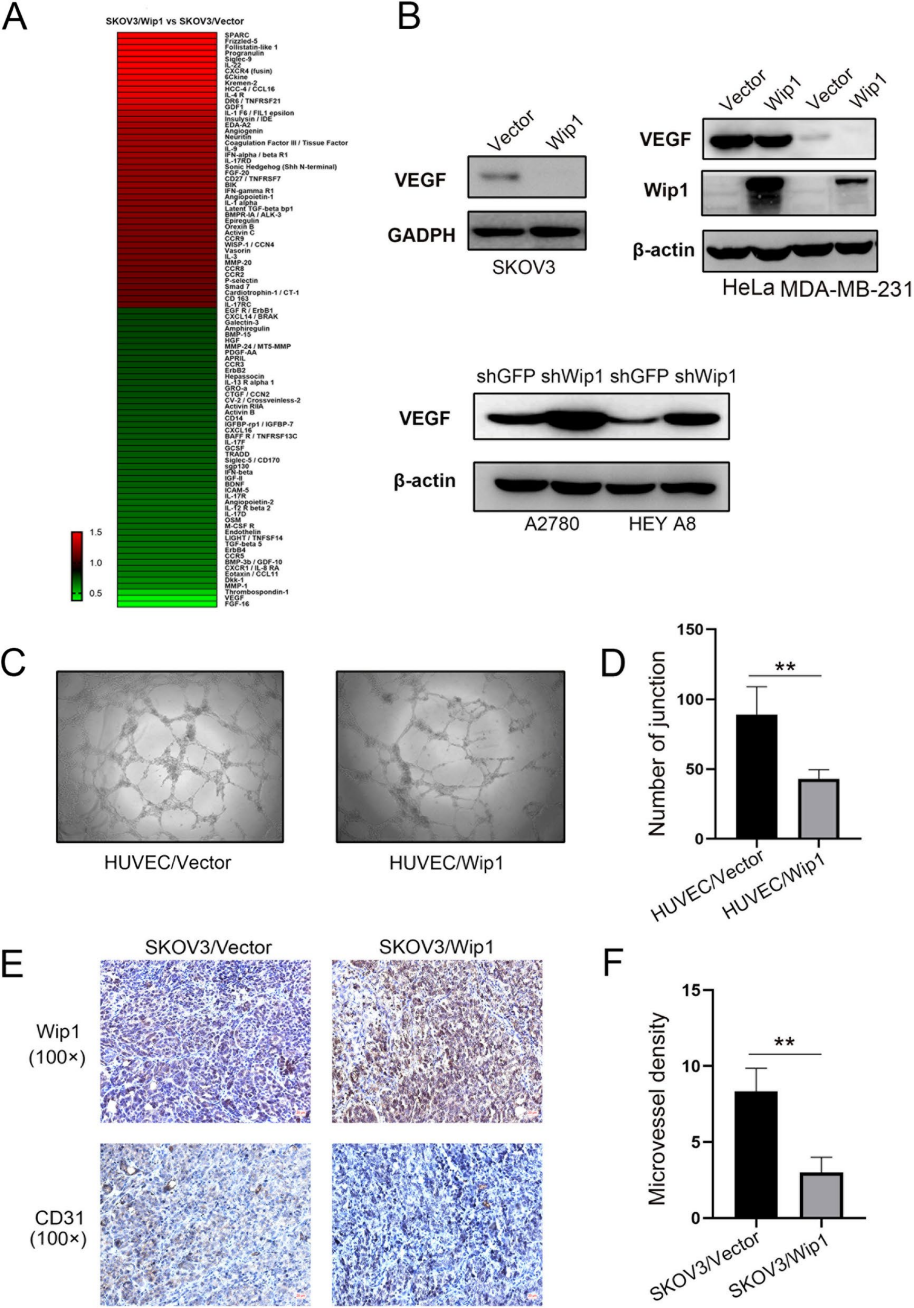
**A** Secretome profiling of differentially expressed cytokines from the culture media of SKOV3/Vector and SKOV3/Wip1 cells. **B** Overexpression of Wip1 decreased VEGF in SKOV3, HeLa and MDA-MB-231 cells, while knockdown of Wip1 increased VEGF in A2780 and HEY A8 cells. **C** Representative images of the tube formation of HUVECs/Vector and HUVECs/Wip1. **D** Statistical analysis of tube branch points. **E** Immunohistochemical staining of Wip1 and CD31 in peritoneally disseminated nodules in nude mice injected with SKOV3/Vector and SKOV3/Wip1 cells. **F** Statistical analysis of microvessel density

The original article has been corrected.
